# Silicon Supplementation Alters the Composition of Herbivore Induced Plant Volatiles and Enhances Attraction of Parasitoids to Infested Rice Plants

**DOI:** 10.3389/fpls.2017.01265

**Published:** 2017-07-19

**Authors:** Jian Liu, Jiwei Zhu, Pengjun Zhang, Liwei Han, Olivia L. Reynolds, Rensen Zeng, Jinhong Wu, Yue Shao, Minsheng You, Geoff M. Gurr

**Affiliations:** ^1^State Key Laboratory of Ecological Pest Control for Fujian and Taiwan Crops, Fujian Agriculture and Forestry University Fuzhou, China; ^2^Institute of Applied Ecology, Fujian Agriculture and Forestry University Fuzhou, China; ^3^Fujian-Taiwan Joint Innovation Centre for Ecological Control of Crop Pests, Fujian Agriculture and Forestry University Fuzhou, China; ^4^Graham Centre for Agricultural Innovation, Charles Sturt University, Orange NSW, Australia; ^5^Zhejiang Provincial Key Laboratory of Biometrology and Inspection and Quarantine, China Jiliang University Hangzhou, China; ^6^Graham Centre for Agricultural Innovation, New South Wales Department of Primary Industries, Menangle NSW, Australia; ^7^College of Crop Science, Fujian Agriculture and Forestry University Fuzhou, China

**Keywords:** HIPV, induced plant defense, biological control, jasmonate, hexanal 2-ethyl, α-bergamotene, β-sesquiohellandrene, cedrol

## Abstract

Silicon (Si) is important in plant defenses that operate in a direct manner against herbivores, and work in rice (*Oryza sativa*) has established that this is mediated by the jasmonate signaling pathway. Plant defenses also operate indirectly, by the production of herbivore induced plant volatiles (HIPVs) that attract predators and parasitoids of herbivores. These indirect defenses too are mediated by the jasmonate pathway but no earlier work has demonstrated an effect of Si on HIPVs. In this study, we tested the effect of Si supplementation versus Si deprivation to rice plants on subsequent HIPV production following feeding by the important pest, rice leaffolder (*Cnaphalocrocis medinalis*). Gas chromatography–mass spectrometry analyses showed lower production of α-bergamotene, β-sesquiohellandrene, hexanal 2-ethyl, and cedrol from +Si herbivore-infested plants compared with -Si infested plants. These changes in plant chemistry were ecologically significant in altering the extent to which parasitoids were attracted to infested plants. Adult females of *Trathala flavo-orbitalis* and *Microplitis mediator* both exhibited greater attraction to the HIPV blend of +Si plants infested with their respective insect hosts compared to -Si infested plants. In equivalent studies using RNAi rice plants in which jasmonate perception was silenced there was no equivalent change to the HIPV blend associated with Si treatment; indicating that the effects of Si on HIPVs are modulated by the jasmonate pathway. Further, this work demonstrates that silicon alters the HIPV blend of herbivore-infested rice plants. The significance of this finding is that there are no earlier-published studies of this phenomenon in rice or any other plant species. Si treatment to crops offers scope for enhancing induced, indirect defenses and associated biological control of pests because parasitoids are more strongly attracted by the HIPVs produced by +Si plants.

## Introduction

Silicon (Si) has not historically been considered an essential plant nutrient though it has been termed ‘quasi-essential’ (Epstein, 1994; Luyckx et al., 2017). Evidence has mounted in the last decade that Si plays important roles in plant defense against biotic and abiotic stress (Ma et al., 2001; Ma, 2004; Ahmed et al., 2013; Balakhnina and Borkowska, 2013; Pontigo et al., 2015; Cooke and Leishman, 2016) including against insect herbivores and pathogens in agriculture (Ye et al., 2013; Wang et al., 2017). Among studies of the effects of Si on plant defense against herbivores, two primary modes of action have emerged as important. The first of these is the mechanical mode afforded by the deposition of inorganic amorphous oxide (SiO_2_) phytoliths in the epidermis of foliage and in spines and trichomes (Ma, 2004; Hartley et al., 2015). These defenses are often constitutive (‘always on’) but can also be induced by damage such that the plant is responding to herbivory with greater deposition of silicon in defense structures (Hartley et al., 2015). Silicon deposition provides structural rigidity to plants and the resulting physical toughness also makes the plant surface less vulnerable to penetration and colonization by fungal pathogens (Wang et al., 2017) and tougher for herbivores to masticate and digest (Kvedaras and Keeping, 2007; Reynolds et al., 2016). The second mode of action of silicon in plant defense is enhancement of the induced production of defense chemicals (Wu and Baldwin, 2010) including enzymes such as polyphenol oxidase and inhibitors such as trypsin protease inhibitor. For both of the foregoing mechanisms, there is strong evidence that the influence of Si is mediated by the jasmonate signaling pathway (Ye et al., 2013). The lipid-derived plant hormone, jasmonate, plays several important roles in plant metabolism including prioritization of defense over plant growth when a plant is under biotic stress (Yang et al., 2012). Ye et al. (2013) demonstrated Si elevated rice plant defense against rice leaffolder (*Cnaphalocrocis medinalis*) by promoting phytolith accumulation in leaves as well as polyphenol oxidase, peroxidase, trypsin protease inhibitor, and Bowman–Birk protease inhibitor activity. The significance of the jasmonate pathway in these effects was established by use of RNAi to silence expression of the CORONATINE INSENSITIVE1 gene which is involved in jasmonate perception. For the RNAi rice plants, the positive effects of Si on plant defenses were negated, leading to normal development of the herbivore. By logical extension, it is expected that the influence of Si on plant defense will also apply to mechanisms other than the two broad types described above, provided that these mechanisms too are mediated by the jasmonate pathway.

Plant defenses are not always direct in nature; they can also operate indirectly via the third trophic level, the natural enemies of herbivores. Work in recent decades has amply demonstrated that plants are able to manipulate the composition of the volatiles they emit, such that they serve as synomones, semiochemicals that benefit both the emitter and receiver (Becker et al., 2015). In this mechanism, a plant responds to herbivore attack by producing a particular herbivore induced plant volatiles (HIPVs) blend to which natural enemies such as predators and parasitoids are attracted, leading them to attack the herbivores (Mumm and Dicke, 2010; Schuman et al., 2012). Crucially, the jasmonate pathway is the most important of the three known signal-transduction pathways that underlie the induction of HIPVs (Ament et al., 2004; Wei et al., 2011; Zhang et al., 2013). Accordingly, the positive effects of Si on direct plant defenses described above may apply to indirect plant defense based on HIPV production.

To date, no work has been published on the effects of Si on HIPVs in any plant system. There is, however, indirect evidence that HIPVs may be affected by Si treatment to plants. Using a cucumber system, Kvedaras et al. (2010) demonstrated that Si enhanced the attraction of the predator, *Dicranolaius bellulus*, to plants infested by *Helicoverpa armigera*. An associated field study showed that Si-treated plants were more attractive to ‘wild’ predators than Si deficient control plants (Kvedaras et al., 2010). Whilst those results are consistent with Si altering the HIPV blend of pest-infested plants, that paper did not include any results for the volatiles emitted by the plants. This leads to the hypothesis that Si alters the HIPV blend thus enhancing the attraction of natural enemies. To test our hypothesis, we studied the volatiles of rice plants grown under Si deficient conditions versus with Si supplementation, and determined the effects on volatiles when infested by a major economic insect pest. We then complemented volatile studies with behavioral assays to determine whether attraction of biologically relevant parasitoid species was affected by changes in volatiles. Finally, RNAi plants were used to explore whether silenced perception affected the influence of the Si regime on plant volatiles.

## Materials and Methods

### Plants

Rice plants of wild type (WT, var. Shishoubaimao) and a CORONATINE INSENSITIVE1 (OsCOI1) RNAi line deficient in jasmonate perception were used in this study. The OsCOI1 line was generated as described by Ye et al. (2013). Plants were hydroponically grown in climate control chambers (27 ± 1°C, 75 ± 5% RH) using a modified Hoagland’s solution (Hoagland and Arnon, 1950). For +Si rice plants, a hydroponic solution with 2.11 mM sodium metasilicate (Na_2_SiO_3_) was used. For -Si rice plants, 4.22 mM of Na^+^ was added to the hydroponic solution to equalize Na^+^ concentrations with +Si rice plants. The pH of solutions was mediated to 5.6–6.0 by adding hydrochloride acid. Hydroponic solution was renewed weekly and plants were grown to 6–8 weeks of age for all studies.

### Herbivores and Parasitoids

Folded rice leaves with middle-aged to mature larvae of *C. medinalis* feeding inside were collected from the experimental farm of South China Agricultural University (23°17′N, 113°36′E). Foliage was kept turgid by immersing the cut ends in water within insect rearing cages (45 cm × 45 cm × 45 cm) placed in climate controlled rooms (27 ± 1°C, 75 ± 5% RH) to obtain adult *C. medinalis*. Newly emerged adults were transferred onto 2- to 3-week-old maize (*Zea mays* L.) plants to generate the larvae used in experiments. The rice leaffolder parasitoid, *T. flavo-orbitalis*, was reared from hosts collected from the experimental farm of Fujian Agriculture and Forestry University (26°29′N, 118°48′E). Army worm (*Mythimna separata* (Walker)) and its parasitoid, *Microplitis mediator* Haliday, were supplied by Keyun Biocontrol (Henan, China) and Hebei Academy of Agricultural and Forestry Sciences (Hebei, China), respectively.

### Plant Volatile Analysis

Rice volatile organic compounds were collected by dynamic headspace collection (Zhang et al., 2009). One rice plant was placed in a 15-L glass vessel. Purified air was pumped into the vessel at 200 mL/min. The system was purged for 1 h before attaching a tube filled with 80 mg matrix Porapak Q (Sigma-Aldrich) to the air outlet to adsorb the volatiles. Headspace collections were carried out at 27 ± 1°C, 70 ± 5% RH, and lasted for 4 h. Samples were collected from plants in each of the following experimental treatments for WT and *OsCOI1* rice: +Si +Herbivore; +Si -Herbivore; -Si +Herbivore; and -Si -Herbivore. All plant treatments were prepared concurrently with individual plants laid out in separate insect proof cages (50 cm × 50 cm × 50 cm). Volatile collection commenced 12 h after the plants were infested with four 3rd instar larvae per plant. Each treatment had nine replicates.

Headspace samples were eluted by 500 μL dichloromethane into 2-mL glass vials, then mixed with 5 μL internal standard nonyl acetate at 100 ng/μL. Volatiles samples were then analyzed by GC-MS (Agilent 7890B-5977A). The temperature was held at 40°C for 3 min then increased at 5°C min^-1^ to 220°C. Compounds were identified by comparing the mass spectra with the instrument’s internal NIST 2011 spectra database and Wiley Spectra Lab (John Wiley & Sons, New York, NY, United States). The quantity of each compound was calculated by comparing the peak area of each compound with internal standard.

### Olfactometer Studies of Parasitoids on WT Rice Plants

Two rice-herbivore-parasitoid systems were used: WT-*C. medinalis*–*T. flavo-orbitalis* and WT-*M. separata–*M. mediator.** To obtain herbivore attacked plants, four 3rd instar larvae of either herbivore species were allowed to feed on one plant for 12 h. All larvae were then removed carefully without further mechanical damage to the plant before connecting to the Y-tube olfactometer. Pairs of plants were set up as: -Si +Herbivore vs. -Si -Herbivore; +Si +Herbivore vs. +Si -Herbivore; and -Si +Herbivore vs. +Si +Herbivore. Each pair of plants was connected to the arms of a Y-tube olfactometer to test parasitoid response to treatments. Plants were placed individually into the volatile collection apparatus and purified air supplied at 100 mL/min. Preference responses of parasitic wasps to the pairs of plants described above were tested in a Y-tube olfactometer. Individual female adult wasps were released at the downwind side of the tube and 2 min were allowed for the wasp to pass the Y-tube junction and remain in that arm, otherwise the wasp was recorded as a no choice. Each odor comparison was repeated on 3–4 days with 10–20 wasps per day.

### Statistical Analysis

Data analysis was undertaken using SPSS software (IBM SPSS Statistics version 24.0, Armonk, NY, United States). For plant volatile analysis, the average elicitation rate (ng/plant/h) was calculated for each identified compound from nine replicates of each treatment (with exception of eight for treatment +Si + Herbivore). Fisher protected least significant difference (LSD) tests of analysis of variance (ANOVA) was used to compare the difference level between treatments.

Y-tube olfactometer data were analyzed using a Chi-square test. Wasps that did not make a choice were excluded from the analysis.

## Results

### Effect of Silicon on HIPVs

**FIGURE 1:**
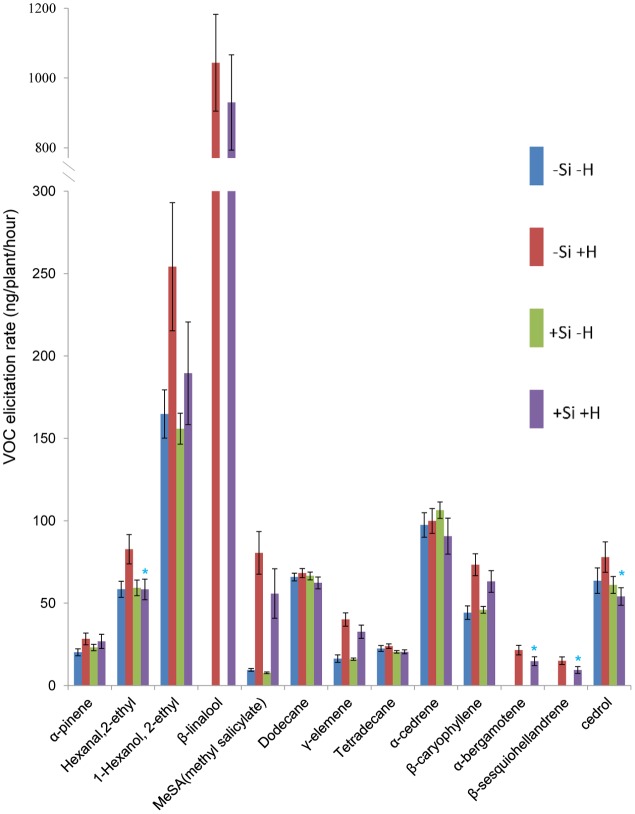
Effect of herbivore (H) (*C. medinalis*) infestation and silicon (Si) treatment on volatile organic compound (VOC) production by wild type rice plants. [Mean ± SE; ^∗^, significant effect of silicon within herbivore treatment (*P* < 0.05) by Fisher’s Least Significant Difference test of one-way analysis of variance (ANOVA)].

**FIGURE 2:**
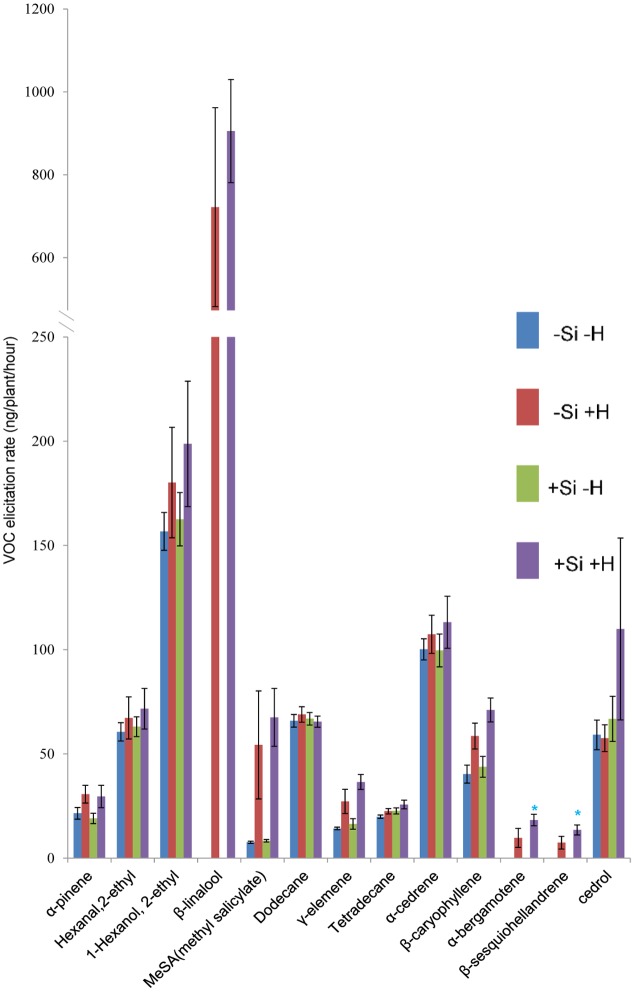
Effect of herbivore (H) (*C. medinalis*) infestation and silicon (Si) treatment on volatile organic compound (VOC) production by *OsCOI1* RNAi rice plants. [Mean ± SE; ^∗^, significant effect of silicon within herbivore treatment (*P* < 0.05) by Fisher’s Least Significant Difference test of one-way analysis of variance (ANOVA)].

Thirteen of 60 compounds were identified from the volatile blend collected as head space samples of rice plants. The composition of volatile blends was affected by *C. medinalis* infestation. For WT plants, β-linalool, α-bergamotene, and β-sesquiohellandrene were detected only in samples from +Herbivore plants, whether +Si or -Si (**Figures 1, 2**). Production of five other compounds, hexanal 2-ethyl, 1-hexanol 2-ethyl, methyl salicylate (MeSA), γ-elemene, and β-caryophyllene, was significantly elevated (*P*-value: 0.011, 0.020, <0.001, <0.001, <0.001, respectively) for -Si +Herbivore plants (82, 254, 81, 40, 73 ng/plant/h, respectively) compared with -Si -Herbivore plants (58, 164, 10, 16, 44 ng/plant/h, respectively). Infested +Si plants produced significantly lower amounts (compared with -Si +Herbivore plants) of four compounds: hexanal 2-ethyl, α-bergamotene, β-sesquiohellandrene and cedrol (**Figure 1**) (58, 15, 9, 54 ng/plant/h, respectively, vs. 83, 22, 15, 78 ng/plant/h, respectively) (*P*-value: 0.013, 0.022, 0.015, 0.027, respectively).

For *OsCOI1* plants, as in WT plants, β-linalool, α-bergamotene, and β-sesquiohellandrene were detected only in samples from +Herbivore plants, whether +Si or -Si (**Figure 2**). Production of MeSA, γ-elemene, and β-caryophyllene was significantly (*P*-value: 0.030, 0.016, 0.020, respectively) elevated for +Herbivore plants compared with -Herbivore plants (-Si +Herbivore: 54, 27, 59 ng/plant/h, respectively, vs. -Si -Herbivore: 7, 14, 40 ng/plant/h, respectively; +Si +Herbivore: 68, 37, 71 ng/plant/h, respectively, vs. +Si -Herbivore: 3, 16, 44 ng/plant/h, respectively) (**Figure 2**). However, the effect of Si on *OsCOI1* plants differed from that observed for WT plants. Infested +Si plants produced higher (rather than lower) amounts of α-bergamotene and β-sesquiohellandrene compared with -Si +Herbivore plants (18, 14 ng/plant/h, respectively, vs. 10, 7 ng/plant/h, respectively) (*P*-value: 0.034, 0.034, respectively); whilst production of hexanal 2-ethyl and cedrol was unaffected by Si treatment (rather than being suppressed by Si as observed in WT plants).

### Parasitoid Response to HIPVs

**FIGURE 3:**
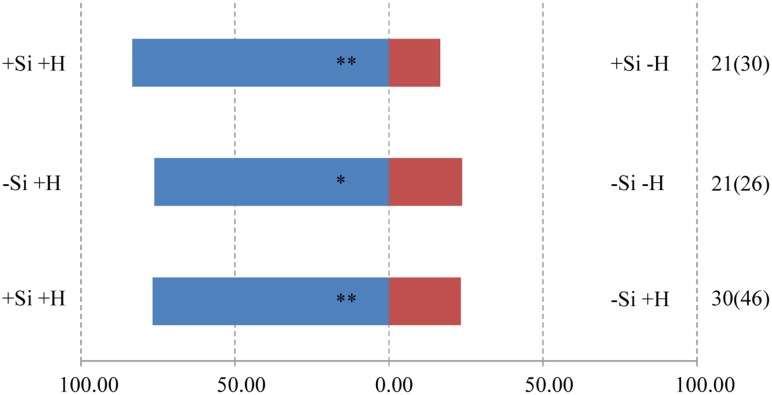
Y-tube olfactometer response of the parasitic wasp, *T. flavo-orbitalis* to volatile profiles of WT rice plants, showing the effect of herbivore (H) (*C. medinalis*) infestation and silicon (Si) treatment. Asterisks indicate significant difference from a 50:50 distribution (Chi-square test; ^∗^*P* < 0.05; ^∗∗^*P* < 0.01; *n*, number of wasp individuals responding to odor source, number in parentheses means the number of wasp individuals used for test). (Statistic comparison, +Si +H vs. +Si –H, χ^2^ = 8.000, *P* = 0.005; –Si +H vs. –Si –H, χ^2^ = 5.762, *P* = 0.016; +Si +H vs. –Si +H, χ^2^ = 8.533, *P* = 0.003). % parasitic wasps to plant odour source.

**FIGURE 4:**
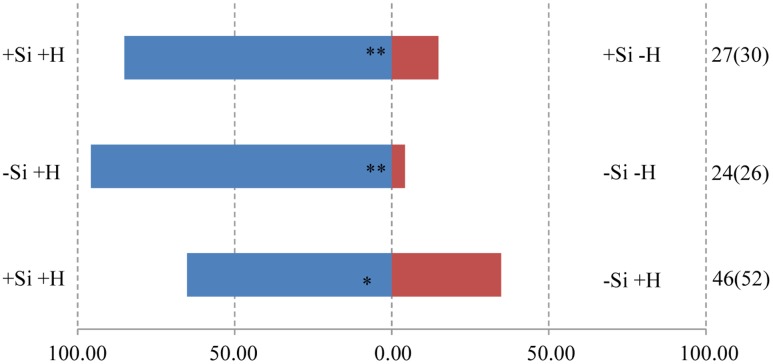
Y-tube olfactometer response of the parasitic wasp, *M. mediator* to volatile profiles of WT rice plants, showing the effect of herbivore (H) (*M. separata*) infestation and silicon (Si) treatment. Asterisks indicate significant difference from a 50:50 distribution (Chi-square test; ^∗^*P* < 0.05; ^∗∗^*P* < 0.01; *n*, number of wasp individuals responding to odor source, number in parentheses means the number of wasp individuals used for test). (Statistic comparison, +Si +H vs. +Si -H, χ^2^ = 13.370, *P* = 0.000256; -Si +H vs. -Si -H, χ^2^ = 20.167, *P* = 0.000007; +Si +H vs. -Si +H, χ^2^ = 4.261, *P* = 0.039). % parasitic wasps to plant odour source.

The responses of both parasitoids *T. flavo-orbitalis* and *M. mediator* to experimental treatments was consistent across species. The volatile blend of +Herbivore plants was significantly more attractive than the blend from -Herbivore plants, regardless of Si regime (**Figures 3, 4**). Both parasitoids also responded significantly more strongly to the volatile blend of +Si +Herbivore than to -Si +Herbivore plants.

## Discussion

Silicon is the second most abundant element in the earth’s crust, yet the vast majority of this is not available to plants (Epstein, 1994; Savant et al., 1997; Ma and Yamaji, 2006), existing predominantly as feldspars and quartz minerals. Plants take up Si from the soil solution as silicic acid [Si(OH)_4_], or Si(OH)_3_O^-^ at high pH, so although the Si content of soils can be as high as 45% (Currie and Perry, 2007), availability can be low. Deficiencies are especially important for plants such as rice that require this element in large amounts (Horuz et al., 2013). Evidence of this is the fact that plants have evolved influx, efflux and channel-type transporters to actively uptake Si (Ma and Yamaji, 2006, 2015; Trembath-Reichert et al., 2015; Vivancos et al., 2016) as well as that yield increases when plant-available Si is added to the growing medium (Tavakkoli et al., 2011). The beneficial roles of Si to various plants have been the subject of extensive research and include alleviation of abiotic stresses such as drought (Marques et al., 2016), metal toxicity, and micronutrient deficiency (Hernandez-Apaolaza, 2014); and biotic stress from pathogens (Winslow, 1992; Vivancos et al., 2015) and herbivores (Reynolds et al., 2009, 2016; Ye et al., 2013). To date, there is increasing evidence of Si priming plants for defense against herbivore attack (Reynolds et al., 2009; Hartley and DeGabriel, 2016). But, a conspicuous gap in knowledge about the effects of Si on plant biology is for the volatiles that are produced by plants and that are often key semiochemicals that drive ecological interactions including induced, indirect plant defense against herbivores enemies (Pare and Tumlinson, 1999; Dicke and Loon, 2000; Degenhardt et al., 2003).

The present results support our hypothesis and show, for the first time in any plant species, that plants grown with available Si have a different HIPV profile compared to plants grown under Si deficiency. Earlier work by Kvedaras et al. (2010) showed that +Si plants were more attractive than -Si plants to a generalist predator in olfactometer tests and to wild predators (of unknown identity) in a field experiment. That study did not, however, include work on plant volatiles so the mechanism underpinning the apparent effects on predators remained unclear. In the present study, several of the volatile compounds that were influenced by herbivore infested rice plants were shown to be HIPVs, since they were not produced by uninfested plants, irrespective of the Si status of the plant. These were β-linalool, α-bergamotene, and β-sesquiohellandrene, each of which have been identified as HIPVs in other studies (Hare, 2011; De Backer et al., 2016; Pinto-Zevallos et al., 2016). Whilst it is possible to record antennal responses for parasitoids to individual HIPVs (Takemoto and Takabayashi, 2015), it is known that blends of HIPVs and the relative quantity of different compounds is more important than any single compound (Van Wijk et al., 2010, 2011). Accordingly, in addition to the three compounds that were produced only by infested plants, several others were produced in greater quantities by infested rather than healthy plants: hexanal 2-ethyl, 1-hexanol 2-ethyl, methyl salicylate, γ-elemene and β-caryophyllene. Most crucially, the differences in blends between plants with a different Si regime (within herbivore treatment) involved a significant change in the composition of volatiles produced, with lower production of two of the HIPVs (α-bergamotene and β-sesquiohellandrene) and of hexanal 2-ethyl and cedrol. Hexanal 2-ethyl (synonym, 2-ethyl hexanol) has been reported as a volatile compound released by *Rutaceae* spp. foliage (Robbins et al., 2012) and is produced by insect-damaged roots of carrot (*Daucus carota*) (Weissteiner and Schütz, 2006). Cedrol has been reported as a foliar and fruit volatile produced by *Ficus carica* L. (Soltana et al., 2017) and, though it has not been reported to be a HIPV, exhibits bioactivity against arthropod pests (Eller et al., 2014).

The foregoing differences in volatile blends between experimental treatments were demonstrated to have ecological relevance by the behavior of two parasitoid wasp species. Both *T. flavo-orbitalis* and *M. mediator* responded more strongly to the volatile blend from +Herbivore plants than -Herbivore plants within each of the Si regimes, demonstrating that the experimental conditions were suitable for these parasitoids to exhibit biologically appropriate behavior consistent with HIPVs guiding them to infested plants. Crucially, both parasitoids also exhibited a significant preference to the odor blend from +Si over -Si when both of these treatments were herbivore-infested.

The nature of the change in volatile blend brought about by Si pre-treatment (**Figures 1, 2**) was not a clear cut switching-on, or -off, for the release of a given compound. Rather, the results are consistent with earlier studies showing that it is the ratio of compounds in blends that is crucial for attraction to natural enemies (Pare and Tumlinson, 1999; Dicke and Loon, 2000; Degenhardt et al., 2003). Specifically, our results are consistent with the heuristic that both parasitoids were attracted to a blend (**Figures 3, 4**) in which the major HIPVs, especially linalool, were present but in which more minor volatiles (hexanal-2-ethyl and cedrol) were at background levels, equivalent to those emitted by herbivore-free plants.

The present study establishes a stronger evidence base for exploring scope to promote biological control of pest herbivores by ensuring that crops have an optimal supply of Si so that they are able to mount a strong induced, indirect defense based on HIPV production. Fully exploiting the apparent priming effects of Si on plant defense demands a more comprehensive understanding of the underlying metabolic pathways. Work by Ye et al. (2013) demonstrated the importance of the jasmonate signaling pathway for positive effects of Si on rice defenses. The present study demonstrates that Si can enhance the attractiveness of the HIPV blends produced by WT rice when attacked by a herbivore but that this effect of Si does not occur among RNAi plants with silenced jasmonate perception. The emission of HIPVs is known to be influenced by the jasmonate pathway, particularly in the case of chewing herbivores such as those used in the present study (Dicke and Baldwin, 2010). Accordingly, the present results support the finding of Ye et al. (2013) that Si promotes plant defenses via the jasmonate pathway, but extends the range of effects to include HIPVs production. Further studies are required to determine whether Si also interacts with the salicylic acid and ethylene defense pathways of plants (Ruther and Kleier, 2005; Zarate et al., 2007; Koornneef et al., 2008; Dicke et al., 2009). Each of these, as well as the jasmonate pathway, can lead to the induction of HIPVs including by feeding of sucking pests such as aphids (Ozawa et al., 2000; van Poecke and Dicke, 2002). It will, therefore, be important to determine the extent of interactions between Si and varying plant defense mechanisms in order to fully exploit the potential of this element in novel plant protection approaches.

## Author Contributions

JL and GG designed the experiments. JL, JZ, LH, JW, and YS conducted the experiment. JL, PZ, and GG analyzed and interpreted the data. JL, OR, MY, and GG drafted and revised the paper. All authors read and approved the final manuscript.

## Conflict of Interest Statement

GG and OR hold research funding from Australian Steel Mill Services (a potential source of silicacious material) and this supports a project being conducted independently of the present work. Australian Steel Mill Services had no role in the present study, interpretation of results or decision to publish. The other authors declare that the research was conducted in the absence of any commercial or financial relationships that could be construed as a potential conflict of interest.
